# Gold-Plated Electrode with High Scratch Strength for Electrophysiological Recordings

**DOI:** 10.1038/s41598-019-39138-w

**Published:** 2019-02-27

**Authors:** Mohaddeseh Vafaiee, Manouchehr Vossoughi, Raheleh Mohammadpour, Pezhman Sasanpour

**Affiliations:** 1 Institute for Nanoscience and Nanotechnology, Sharif university of Technology, Tehran, Iran; 2 Department of Chemical and Petroleum Engineering, Sharif University of Technology, Tehran, Iran; 3 Institute for Biotechnology and Environmental (IBE), Sharif University of Technology, Tehran, Iran; 4 Department of Medical Physics and Biomedical Engineering, School of Medicine, Shahid Beheshti University of Medical Sciences, Tehran, Iran

## Abstract

Multi electrode arrays (MEA) have been exploited in different electrophysiological applications. In neurological applications, MEAs are the vital interfaces between neurons and the electronic circuits with dual role; transmitting electric signal to the neurons and converting neural activity to the electric signal. Since the performance of the electrodes has a direct effect on the quality of the recorded neuronal signal, as well as the stimulation, the true choice of electrode material for MEA is crucial. Gold is one of the best candidates for fabrication of MEAs due to its high electrical conductivity, biocompatibility and good chemical stability. However, noble metals such as gold do not adhere well to the glass substrate. Consequently while exposing to the water, gold films are damaged, which impose limitations in the exploiting of gold thin films as the electrode. In this paper, a simple and cost effective method for the fabrication of gold electrode arrays is proposed. Using various mechanical (adhesion test and scratch strength), morphological (AFM and SEM) and electrochemical methods, the fabricated electrodes are characterized. The results show that the fabricated electrode arrays have significantly high scratch strength and stability within the aqueous medium. In addition, the electrical properties of the electrodes have been improved. The proposed electrodes have the potential to be exploited in other applications including electronics, electrochemistry, and biosensors.

## Introduction

The brain and its fundamental part, nerve cells, play an important role in the life of beings. Therefore, the study of the system has been widely considered. For studying the nervous system, various methods have been presented. One of the most commonly applied methods is multi-electrode arrays which are used to study the function of the cell and the neural tissue^1–3^.

MEAs have been used *in vitro* to stimulate the cells and record the extracellular activity of single cells and electrogenic cellular networks^4,5^ and thin tissue sections such as hippocampus^6–9^ and to treat or improve damaged brain tissues or peripheral nerves. MEAs are useful tools for the study of electrogenic cells^10–12^. These electrodes consist of a number of arrays that lie next to each other on a flat bed. Although extracellular recording suffers from low signal-to-noise ratio (SNRs), minimal invasion allows long-term recording, and multi-microelectrode capabilities allow large-scale studies to be conducted^13^. Because of being non-invasive and keeping cells alive, this method is suitable for long-term tests. In addition, MEAs can detect the electrical signals of cells from multiple points. Another advantage of this method is the possibility of simultaneous recording and stimulation, so that the array can be used to stimulate the electrical system and at the same time recording. These features make possible the study of neural networks that have a complex structure.

A typical microelectrode array consists of a set of metal electrode, insulating layer for working in liquid medium, and connections to external electronic unit^14,15^. In other words, the structure of the electrode consists of three main parts: the exposed electrodes, the paths and the connection pads. In this regard three layers of substrate, conductive layer and insulation layer are required. Usually the substrate is made up of an insulating material such as glass and the rest of the layers are placed on it. The next layer, (conductive layer), is the main part of the electrode which transmits signals of the cells. Finally, the insulation layer as an obstacle, isolates the contact of the rest part of the conductive layer with the cells and media. The significant factors that are considered for selecting materials include biocompatibility, optical transparency, stiffness and durability of insulating and conducting layers under cell culture conditions^16^.

Typically for manufacturing of MEA devices, various metals are employed including gold^17–22^, titanium^23^, electroplated platinum black^24–28^, tungsten^20^, platinum^29–32^, and iridium^27,33^ which are deposited on the silicon, glass or various flexible substrates. Gold is well known for its biocompatibility, high conductivity and high corrosion resistance and therefore is the ideal choice for this application^16^. On the other hand noble metals such as gold and silver do not adhere well to the glass substrate and films are damaged while exposed to the water which restricts its application^34^. Considering the stability of the electrode, it is necessary to provide appropriate adherence of gold to the substrate, to prevent electrode damage during the experiment. The most common way is to deposit a thin layer of titanium or chromium on the substrate which improves the adhesion of gold to the substrate^34,35^. However, the potential cytotoxicity of these adhesive layers and their oxides leads to strong demand for alternative adhesive layers^36,37^.

Another challenge for improving the performance of electrodes is increasing the area of the electrochemically active surface without increasing the geometric surface of the electrodes. On the other hand recording electrodes should have small impedance to achieve low noise and high signal-to-noise ratio. A parameter that affects SNR is the electrode noise (mainly thermal noise). As a result, the amplitude of the noise can be reduced by decreasing the impedance of the electrode. Since the impedance is mainly capacitive, this can be achieved by increasing the area of the electrochemical effective surface area^38^. Reducing the size of the electrodes also enables the placement of several electrodes in a high density array which creates higher spatial resolution. On the other hand, the evaluation of cellular function over a long period of time is important for monitoring the physiological processes, such as the destruction of nerve tissue that occurs, for example, in Alzheimer’s disease^14^. Therefore obtaining a stable electrode array for long-term function without change in the properties of the electrode is another challenge facing this area.

Here in this research, we have addressed the mentioned challenges in MEA technology. The main goal is the achievement of gold electrode with high stability in the biological environment while keeping robust structural and steady functional properties over times of usage. As a double-edged sword, gold based MEAs are expensive, while they exhibit brilliant properties. By reducing the costs of fabrication and improving the lifetime and mechanical properties (such as high scratch strength and stability within the aqueous medium) the advantageous will dominate. In this regard, by introducing an easy-access method, the adhesion of gold layer to the glass substrate has been improved and a novel method for fabrication of MEA has been presented. The presented method is much simpler and less costly compared to traditional methods and can be employed in large scale area which potentially can be applicable for fabrication of integrated multielectrode arrays. The surface modification is also effective in improving the strength of the electrode surface against scratches. In this way, a stable electrode has been obtained for long-term functions with low impedance. The stable conductive electrodes attained through this method have potential applications in other fields such as connectors, for optoelectronic devices and photovoltaics and various types of electrochemical and biological sensors.

Characteristics of the electrode were examined employing electrochemical, adhesion and scratch tests. Finally, in order to check the functionality of the electrode, neural signals from the mouse brain have been recorded. Overall, the results of our study suggest that the proposed method of fabrication could be used as an effective method for improving the adhesion of gold layer to the glass substrate in electrodes used in biological applications. The method offers significant adhesion comparing with chromium, superior electrical properties, remarkable mechanical stability in biological fluids and high scratches strength.

## Results

### Roughness measurements of the electrode surface

**Figure 1:**
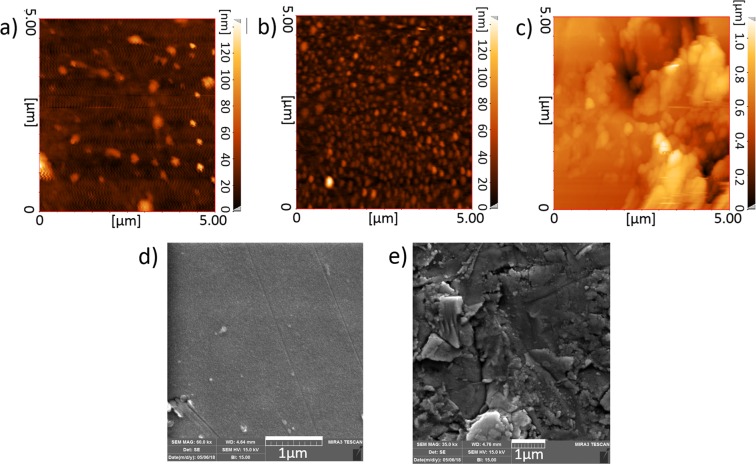
AFM topography image of the electrode surface. (**a**) HF-Sputt, (**b**) Sputt, and (**c**) Rough-Sputt, and SEM image of the electrode surface (**d**) Sputt, and (**e**) Rough-Sputt.

Figure 1a–c shows the AFM topography image of the surface of three samples (HF-Sputt, Sputt, and Rough-Sputt). The random ups and downs on the surface of electrodes are seen in the topographic images. It has been shown that mechanical and chemical processes inherently generate random roughness on surfaces^39^. The surface of the HF-Sputt sample is somehow smoother comparing with two others. The proposed improved method (Rough-Sputt sample) shows the highest surface roughness. Also Fig. 1d,e which are the SEM images of the surfaces of Sputt and Rough-Sputt electrode, illustrate the smoothness and roughness of the surfaces of the Sputt and Rough-Sputt electrode, respectively.

The roughness of a surface is typically demonstrated by domain parameters such as the mean surface roughness. The mean values of the roughness of the surface are 7.11, 7.51 and 117 nm for HF-Sputt, Sputt and Rough-Sputt samples, respectively. The roughness ranges of all three electrodes are in nm range. The mean value for Sputt and HF-Sputt electrode are somehow similar. The HF-Sputt electrode has the least mean value of roughness, and Rough-Sputt has the highest.

The difference in the roughness observed in three samples is due to the diverse preparation methods. The Sputt sample includes thin film deposition of the gold layer on the glass substrate without any surface pre-modification. Therefore, the surface is not smooth and has roughness. For the HF-Sputt sample, the glass slip has been placed in a HF solution before the deposition. The HF solution has the ability to etch the glass^40^. Due to the short duration of etching, no significant change was found in the surface roughness. As a highly corrosive liquid, hydrofluoric acid is a harmful carcinogenic and poisonous material for exposure of skin or eyes, or when inhaled or swallowed^41^. In this regard, the HF free techniques are recommended. For the Rough-Sputt sample, the surface of glass slip is mechanically roughened by means of Silica powder before deposition, where the surface of sample will find high roughness.

### Investigate the adhesion of the gold layer

Since the gold layer is the strategic part of the electrode structure, any damage to this layer will lead to variation of conductivity and sometimes disconnection. In this study, with the help of surface roughness, we have sought for improving the adhesion between the conductive layer and the glass substrate of the electrode. In this regard, the chance of damage, scratches and detachment of the conductor layer at the electrode surface has been minimized and, as a result, the stability and lifetime of the electrode with stable properties will be increased. Ultrasonic and tape test, have been applied to study the adhesion of the gold layer to the glass substrate.

#### Tape test

**Figure 2:**
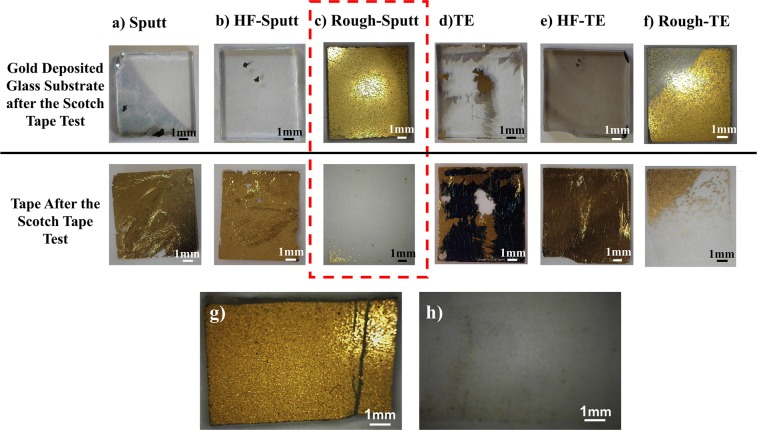
Image of the Au-coated glass substrate and tape after the scotch tape test. (**a**) Sputt (**b**) HF-Sputt (**c**) Rough-Sputt (**d**) TE (**e**) HF-TE, and (**f**) Rough-TE; Image of (**g**) the Au-coated glass substrate and (**h**) tape after the scotch tape test by applying a force of 10 kg.

Figure 2, shows the images of the electrode surface and tape for different electrodes after the test. As shown in the Fig. 2, samples with unmodified glass substrate surface are easily lifted after the adhesion test and almost most part of the gold film is transferred to the tape (Fig. 2a,d). For the samples treated with HF, the same result is observed (Figure 2b,e). Considering the mechanically roughed samples, tiny amount of gold has been transferred to the tape, which indicates better adhesion of gold to the glass surface (Fig. 2f,c). In fact, modified substrates have shown the strongest adhesion. Comparing the method of deposition, the sample deposited by the thermal evaporation method shows lower adhesiveness than the sputtered sample (Fig. 2f).

In Fig. 2g,h, the sample surface and the adhesive isolated from the Rough-Sputt electrode surface is observed after the tape test with different heavier condition (10 kg, and 10 min). As shown in Fig. 2g, despite the high applied force, the surface of the electrode retains the uniformity of gold layer. On the other hand, very small amount of gold has been transferred to the adhesive surface. Comparing the Fig. 2g,h, it seems that the most of the gold is transferred in the part where the groove is created on the surface of the gold and the edges of the electrode. It can be concluded that the transfer of gold in these areas is due to the presence of gold isolated from the surface during the formation of the groove and the lack of proper connection to the edges of the sample.

#### Ultrasonic test in liquid medium

**Figure 3:**
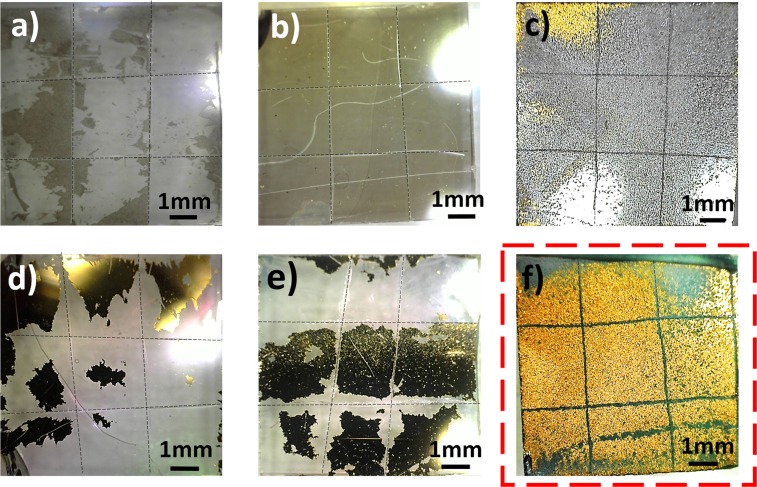
Sample surface image after applying ultrasonic test. (**a**) TE (**b**) HF-TE (**c**) Rough-TE (**d**) Sputt (**e**) HF-Sputt, and (**f**) Rough-Sputt.

Figure 3 shows the surface of the electrodes after the ultrasonic test. As it can be seen, the highest amount of gold remains on the surface of the Rough-Sputt sample. In HF-TE and TE specimens, after immersion in DI water and even without ultrasonic application, the gold layers have been already separated from the substrate. This shows the low adhesiveness of these specimens, especially in liquid conditions. Comparing the images, samples deposited by thermal evaporation, have a slight amount of remaining gold on the surface. While for the structures deposited with sputtering method, the amount of remaining gold is considerable. It seems that comparing with thermal evaporation method, sputtering technique provides better adhesion of layer to the glass substrate. As mentioned before, for the thermal evaporation, a thin layer of Chromium has been deposited to improve the adhesion of the gold layer to the glass substrate. Some portions of the remaining Chromium layer are observed in the Fig. 3a–c.

The results of two experiments have proven that the roughened glass substrate deposited with sputtering method would provide a layer with significant adhesion to the surface.

#### Investigating the scratch strength of electrodes

The maximum applied load of 5N, did not produce any scratches on the sputtered electrodes. This indicates the scratch strength of the sputtering. The electrodes prepared with thermal evaporation method (TE) had less scratch strength. The scratch strength for HF-TE, TE and Rough-TE specimens was 2.8, 3.2 and 3.4 GPa, respectively.

**Figure 4:**
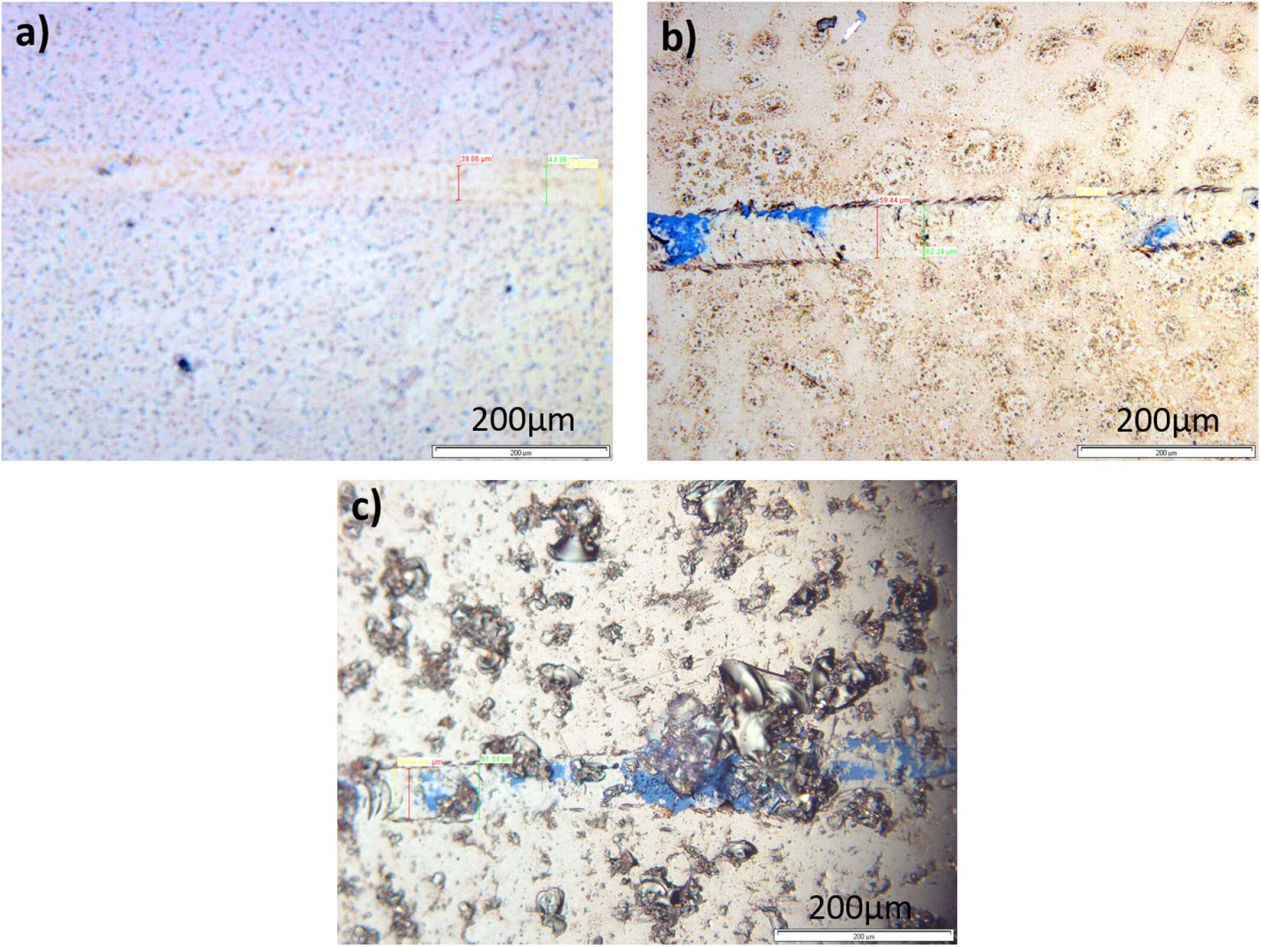
The image of samples after scratch tests. 2N force on (**a**) HF-TE electrode, 5N force on (**b**) TE electrode and (**c**) Rough-TE electrode.

Figure 4 shows the surface image of three electrodes after scratch tests, where the scratches formed on the surface of the electrodes can be observed. According to Fig. 4 and test results, the Rough-TE electrodes show the highest scratch strength compared with HF-TE and TE. The smallest amount of scratch hardness is for the HF-TE structure. As shown in Fig. 4a, the scrubbing needle has softly created a groove with an average thickness of 41 micrometers on the surface of the HF-TE sample. Strength against scratch prevents external connection damages and consequently, keeps the electrical properties of the electrode constant. The stability of the electrode properties over time is an important parameter for identical and comparable recording results. On the other hand, it increases the longevity and stability of the electrode, which is also important from economical point of view.

### Electrochemical Characterization of the Electrodes

The electrochemical properties of the Sputt and Rough-Sputt electrodes have been investigated using CV and EIS.

#### Spectroscopy of electrochemical impedance for electrodes

**Figure 5:**
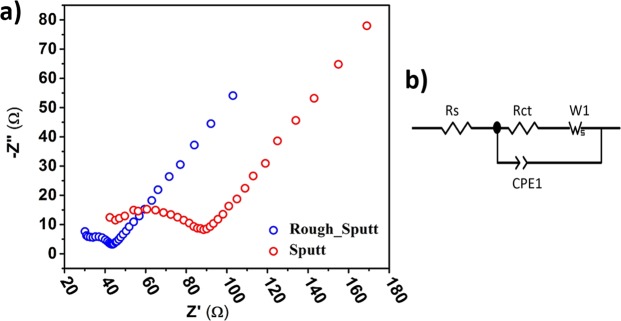
(**a**) EIS Nyquist plot of Rough-Sputt and Sputt electrodes. (**b**) Randles circuit.

Electrochemical impedance spectroscopy have been used to study the interface of the electrolyte-electrode Sputt and Rough-Sputt electrodes. Electrodes with a high surface area (0.8 cm^2^) were made to obtain repeatable and stable results. The Nyquist plot of EIS for electrodes (Rough-Sputt and Sputt) is shown in Fig. 5a. The graphs show that the Rough-Sputt electrode, despite the equivalent area with the Sputt electrode, has lower impedance.

The equivalent circuit of the electrode-interface can be simulated with the Randles circuit model shown in Fig. 5b. The equivalent circuit generally refers to three main elements; the solvent resistance, the double layer capacitance of electrode interface and the charge transfer resistance. The proportional values for each electrode (obtained with the ZView software) are given in Table 1.

**Table 1:** Proportional quantities for each electrode obtained from the Randles model.

	Rough-Sputt	Sputt
Rs	23.46	34.51
Rct	19.51	50.79
CPE1-T	6E-5	3.9082E-5
CPE1-P	0.66579	0.66838
W1-R	482.2	1984
W1-T	72.76	699.2
W1-P	0.4699	0.46862

Given that the value of R_Ct_ is descriptive of the charge transfer rate, it is observed that this value for the Rough-Sputt modified electrode is lower, which indicates the faster rate of transfer of charge than the Sputt electrode. Thus, by improving the electrode surface and lowering the charge transfer resistance, the performance of electrode will be increased. The combination of a small charge transfer resistance with a large interface capacitance for Rough-Sputt modified electrode could be advantageous in suppressing noise and is probably beneficial for stimulation^42^. The constant phase element (CPE) with values of 60 μS·s^0.66579^·cm^−2^ and 39 μS·s^0.66838^·cm^−2^ for the Rough-Sputt and Sputt electrodes respectively, is proportional to the value of the capacitance of the double-layer of range of 1–10 μF·cm^−2^ ^43–45^. Diffusion can create an impedance known as the Warburg impedance. This impedance depends on the frequency of the potential perturbation. At high frequencies the Warburg impedance is small since diffusing reactants don’t have to move very far. At low frequencies the reactants have to diffuse farther, thereby increase the Warburg impedance. The Warburg impedance is generally negligible at high frequencies because the time interval is too short to be sufficient for the ion diffusion reacted on the electrode surface^46–48^.

#### The cyclic voltammetry investigation

**Figure 6:**
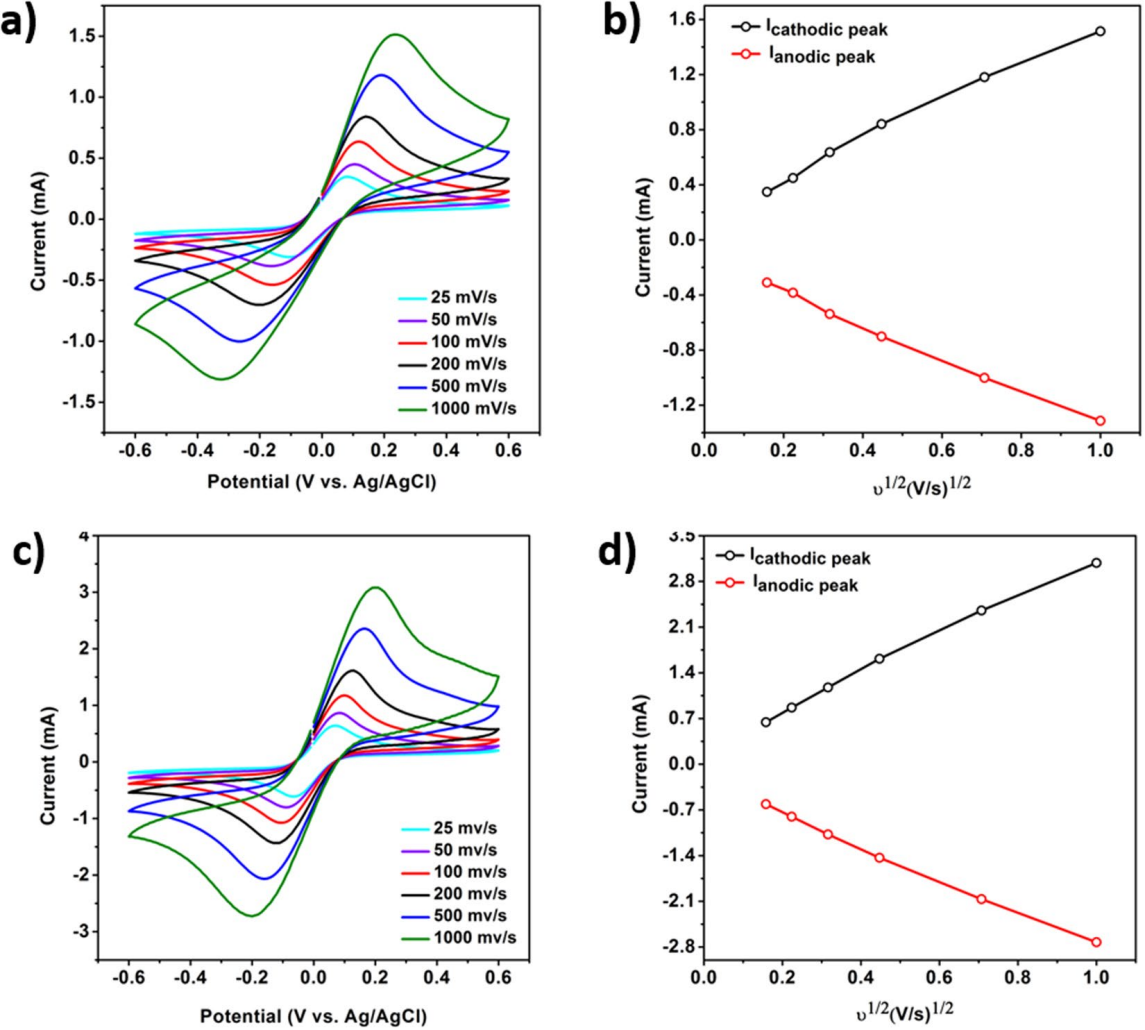
(**a**,**b**) Cyclic voltammetry with different scan rates for the Sputt gold electrode (simple). (**c**,**d**) A cyclic voltammetry with different scanning rates for the Rough-Sputt gold electrode (roughened surface).

The recorded cyclic voltammetry for Sputt and Rough-Sputt electrodes at different scanning rates from 25 mV/s to 1 V/s is shown in Fig. 6a,c. Figure 6b,d show the plot of the peak of the anodic/cathodic flows vs. the square root of the sweep rate. As shown in Fig. 6, for the same applied voltage range to both electrodes, the Rough-Sputt electrode will find a greater current than the Sputt electrode. Receiving more current by applying constant voltage is one of the benefits of electrodes, which will results in noise reduction, as well as reducing the damage to the cells and biological tissues. In accordance with the diagrams, the Rough-Sputt electrode boosts current approximately twice as much as the Sputt electrode. This result is in accordance with the EIS results.

The effective surface area of electrodes can be calculated based on the CV diagram^47,49^. Based on the values of the correlation constant at a cyclic rate of 25–1000 mV/s, 0.0012 and 0.0014 (anodic and cathodic sweep) for Sputt electrodes and, 0.0025 and 0.0029 (anodic and cathodic sweep) for Rough-Sputt electrodes, the effective surface area of 78.2 mm^2^ and 37.8 mm^2^ for the Rough-Sputt and Sputt electrode has been calculated respectively. By comparing the area of the electrodes, it is observed that by increasing the roughness of the surface of the Rough-Sputt electrode, the effective surface area is increased. The difference in area and effective surface in the Sputt electrode is also related to the early roughness of the glass surface, which is also observed in the AFM images.

### Electrophysiological records

**Figure 7:**
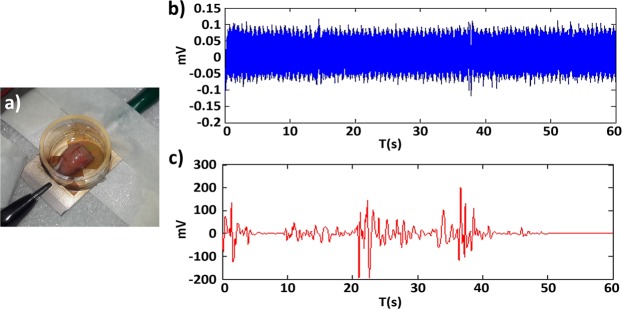
(**a**) Mouse’s brain on the electrode. (**b**) The recorded signal without the brain (Amplified). (**c**) The recorded signal from brain activity (Amplified).

In order to show the functionality of the Rough-Sputt electrode, we have used the electrode structure to measure the local field potential. In this regard, as show in Fig. 7a, the mouse brain has been placed in an artificial cerebrospinal fluid solution on electrodes’ surface and the potential has been measured accordingly. The signal level (comparing with the noise level) has been in the appropriate range.

The recorded signals of mouse brain with the Rough-Sputt electrodes are shown in Fig. 7b. Based on the recorded signal of the neural activity, the electrode is functioning properly and is able to capture the local field potential of mouse brain. As the local field potential measurements show, the electrode structure is able to transfer the signal with an acceptable level (comparing with the signal in the absence of the brain slice).

## Discussion

A novel, low cost, easy to implement method for fabrication of multi electrode array of gold structure has been presented and the performance of fabricated biocompatible electrodes has been evaluated. Based on the results of various mechanical and electrical analysis on the fabricated electrodes, it can be concluded that the mechanical modification of glass substrate before deposition, will be resulted in the electrode structures with excellent properties. On the other hand, excluding the deposition of adhesive layer, is affordable in terms of time and costs and improves the biocompatibility of the electrode in turn.

The surface modification with roughened structure has great potential for cell adhesion. Scratch strength, mechanical stability, improved adhesion of gold layer, and small value of charge transfer resistance as the most important advantageous of our fabricated electrodes have been evaluated by different mechanical, electrochemical and electrophysiological measurements. Utilizing glass as a transparent substrate, make the fabricated electrodes applicable along with microscope, while their robust properties make them operational in the incubators. Since the pattern of the electrode has been created by laser scanning, the lithography process (photoresist deposition, baking, exposure, and development) is not required. In addition, as the surface of the electrode is not exposed to any solvent, photoresist or electroplating bath, the whole process is clean and toxic free. Due to the roughness of the electrode surface, this method is not suitable for the applications that require a smooth surface. In these applications, by increasing the thickness of the gold layer and smoothing the surface after deposition, the robust smooth electrodes could be obtained.

Overall the presented method is much simpler and less costly compared to traditional methods and can be employed in the large scale fabrication. Also in addition to improving the adhesion of gold layer to the substrate, the technique brings more advantageous including: increasing surface roughness, increasing cell adhesion to the surface, high scratch strength and stability of electrodes. The fabricated electrodes and proposed method of fabrications can be exploited not only for multi electrode arrays in neural engineering applications, but for various applications in biosensors and various types of electrochemical based sensors.

## Methods

### The electrode fabrication process

**Figure 8:**
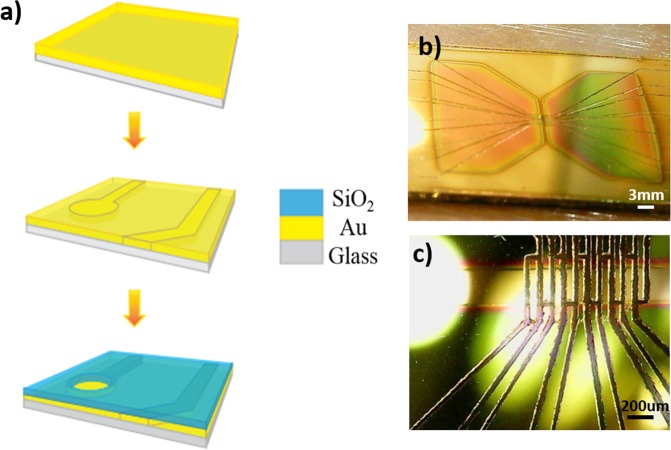
(**a**) Multi electrode array fabrication steps. (**b**) The fabricated electrode surface. (**c**) The contact area of the electrode.

As illustrated in Fig. 8a, the fabrication of electrodes is composed of three main stages: 1-Thin film deposition of gold, 2-Patterning the surface of gold layer, 3-Deposition of insulating layer of SiO_2_. In order to provide the transparency (further monitoring of the biological species with microscope), we have selected glass as the substrate. In order to improve the adhesion of gold layer to the glass substrate, the substrate has been modified initially. In this regard, the surface of glass substrate has been roughened with nanoscale ripples. For this purpose the glass substrate of the electrode is applied on a stone surface covered with silica powder. The roughed surface of glass substrate will be deposited with gold layer. In addition to improving the adhesion of gold layer to the substrate, surface modification brings more advantageous including: increasing surface roughness, increasing cell adhesion to the surface, scratch strength and stability of electrodes. 100 nanometers of gold layer has been deposited on the modified surface using sputtering (Desk Sputter Coater-DST3-A, Nanostructured Coating Co.) and thermal evaporation (Edwards) technique.

In the second step, in order to transfer the pattern of the electrode structure on the surface of gold layer, we have used direct laser writing method. In this regard, the surface of gold layer is irradiated with the laser YW-300B (power 20 W, wavelength 1.06 um, frequency 50/60 Hz) and the pattern of the electrode is transferred on the surface of gold point by point. Considering different contact points (cell-electrode contact and electrode-circuit contact) and electrical routes (conductive paths), three different regions are required to be patterned. The most important part of the MEA structure is the region where the electrode surface is in contact with the neuron cells (active region). Based on the different geometry of the active region (rectangular, and spherical), the equal surface area 200 * 100 µm^2^ has been considered for the region accordingly. The distance between the electrodes has been considered 200 µm (Fig. 8b). The pattern and spacing of the electrodes depend on the application and are limited by the fabrication process constraints.

The third step is the deposition of insulating layer. In order to limit contact area between the electrode and the cell membrane, conductor lines should be deactivated by an insulating layer. A good insulation layer will minimize the signal interference and attenuation and will improve the signal to noise ratio accordingly. In addition, as the electrode is in direct contact with aqueous solutions containing various ions, an inert insulation layer is required to maintain any degradation. Based on this fact that the insulation layer acts as a cell culture substrate, the selected material should be biocompatible^50^. In this regard, SiO_2_ has been considered to insulate the electrode paths. For this purpose, based on the geometry, the appropriate stainless steel masks were made using Lasers and by placing the mask on the surface of electrode, the SiO_2_ layer has been deposited on the electrode using an RF sputtering method. 200 nm thickness of SiO_2_ has been deposited on the surface as insulating layer. Figure 8b shows the fabricated electrode surface including gold thin film and SiO_2_ insulating layer. As shown, some parts of the electrode consist of pads and the contact area are intact (without insulating layer).

Finally, as it is required to provide growth medium and required buffer solutions together with the biological part, it is necessary to arrange for a chamber on the electrode in order to contain the solution parts. The chamber should be fully attached to the surface of the electrode in order to provide proper sealing between the electrode and the enclosure. For this purpose, glass cylinders of 3 cm diameter has been used as the chamber. Poly-dimethyl-siloxane polymer (PDMS) has been applied to attach the chamber to the electrode surface accordingly.

Since the pattern of the electrode has been created by laser scanning, there is no need for any further lithography process (photoresist deposition, baking, development) and the cost of the electrodes will be much lower while the process is easy to implement in each laboratory. In addition, as the surface of the electrode is not exposed to any solvent, photoresist or electroplating bath, the whole process is clean and toxic free.

### Investigation the gold adhesion to the glass substrate

One of the main goals of our study is improving the adhesion of the conductive layer to the substrate, which is performed by roughening of the electrode surface and thereby increasing the stability of the electrodes. We have checked the adhesion of gold layer to the glass substrate using two methods of ultrasonic exposure and tape test. The test has been performed on three samples with different modifications. The first sample includes thin film deposition of the gold layer on the glass substrate without any surface pre-modification. For the second sample, in order to etch the surface of substrate, the glass slip has been placed in a HF solution for 30 seconds. After etching with HF, the slip has been immersed in DI water and has been dried with nitrogen. After preparation of etched substrate, the gold layer will be deposited (HF sample). For the third sample, the surface of glass slip is mechanically roughened by means of Silica powder and gold thin film will be deposited after that (Rough sample). In order to find the best method for efficient adhesion between the conductive layer and the substrate, the deposition for all three samples has been performed with two different methods; sputtering and thermal evaporation. Since the presence of Chromium or Titanium layer between the glass substrate and the gold layer improves adhesion, for the thermal evaporation samples, about 10–15 nm of chromium layers has been deposited on the surface before deposition of gold layer. Finally two groups of samples, sputtered (Sputt, HF-Sputt, and Rough-Sputt) and thermal evaporated (TE, HF-TE and Rough-TE), have been prepared accordingly.

#### Tape test

To determine the adhesion of the gold layer to the glass substrate, a qualitative simple test known as the Scotch tape test has used^51,52^. In this test, first a piece of Scotch tape is attached to the electrode surface (1 cm^2^) and pressure of 67 kPa will be applied for 1 minute. In order to apply a uniform pressure on the tape and the electrode, a glass plate has been placed between the Scotch tape and the weight (670 mg). After that, the tape will be removed from the electrode surface. Depending on the adhesion of the gold film to the electrode surface, a fraction of the gold film is transmitted to the tape. Visible damage on the isolated Scotch tapes will provide an indication of adhesiveness.

In order to further investigation of the adhesion of the gold layer in the Rough-Sputt sample, a tape test with a higher amount of force and more loading time was performed. In addition a groove was created on the surface of the electrode for further examination. Then, in order to carry out the test, a piece of adhesive was attached to the sample surface and a weight of 10 kg was placed on the tape and sample for 10 minutes.

#### Ultrasonic test in liquid medium

Given that the electrode is used in liquid, such as cell culture media or artificial cerebrospinal fluid, an ultrasound test has been designed to examine the adhesion of the gold layer in the liquid medium. In order to perform the test, the grooves have been created on the gold surface. The grooves act as active regions for removing the gold layer from the substrate. The samples have been immersed in 20 ml of deionized water and placed in the ultrasonic bath for 10 minutes.

### Investigation of the scratch strength of electrodes

In order to verify the scratch strength of the electrodes, a scratch test has been performed based on the ASTM G171-03 standard. A diamond scratcher needle, cone shaped with a radius of 200 μm and a tip angle of 120° has been used, derived with the speed of 1.5 mm/sec on the samples, and the load of 2 and 5 N have been applied.

The scratch hardness number is calculated by dividing the applied normal force on the stylus by the scratch width. Equation 1 shows the scratch hardness.

1 $$H{S}_{P}=8P/\pi {w}^{2}$$

where HS_P_ is scratch hardness number, P is normal force, and w is scratch width^53^.

### Roughness measurements of the electrode surface

In this study, the atomic force microscope (DME Nano Technology GmbH, DS 95 Series) was used to measure the surface roughness of the three types of gold-based electrodes (HF-Sputt, Sputt, Rough-Sputt). Before AFM imaging, all samples were washed in acetone and then dried with nitrogen in order to remove any organic and dust contamination.

### Electrochemical characterization of electrodes

In order to find the behavior of electrodes in the biological solutions and buffers, electrochemical properties of electrodes have been investigated using cyclic voltammetry (CV) and electrochemical impedance spectroscopy (EIS) in ferrocyanide solution containing 0.1 M KCL with 0.01 M [Fe(CN)_6_]^4−/3−^. In this regard, the fabricated array of electrodes are considered as working electrode, the Ag/AgCl electrode serves as the reference electrode and a platinum wire is the counter electrode. CV and EIS have been performed on two sputtered samples; the normal substrate (Sputt) and the roughened substrate (Rough-Sputt).

### Electrophysiological records

In order to evaluate the electrode function in recording situation, we have measured the electrical activity of the mouse brain *ex-vivo*. The experiments were performed on rat (9 weeks). The rat was anesthetized using ambient vaporization of ether-soaked gauze. The brain was then removed by surgery on the rat head. The brain was placed inside an artificial cerebrospinal fluid inside the electrode chamber at room temperature for recording not later than ten minutes. All surgeries were conducted under anesthesia and the maximum efforts were performed to minimize suffering. All animal care procedures were performed according to international guidelines on the use of laboratory animals and were approved by Tarbiat Modares university ethical committee for animal research.
